# Intense femtosecond optical pulse shaping approaches to spatiotemporal control

**DOI:** 10.3389/fchem.2022.1006637

**Published:** 2023-01-12

**Authors:** Debabrata Goswami

**Affiliations:** ^1^ Department of Chemistry, Indian Institute of Technology Kanpur, Kanpur, India; ^2^ Center for Lasers and Photonics, Indian Institute of Technology Kanpur, Kanpur, India

**Keywords:** femtosecond pulse shaping, pulsed optical tweezers, coherent control, kerr effect, thermal lens spectroscopy, convection, microheterogeneity, interface

## Abstract

For studying any event, measurement can never be enough; “control” is required. This means mere passive tracking of the event is insufficient and being able to manipulate it is necessary. To maximize this capability to exert control and manipulate, both spatial and temporal domains need to be jointly accounted for, which has remained an intractable problem at microscopic scales. Simultaneous control of dynamics and position of an observable event requires a holistic combination of spatial and temporal control principles, which gives rise to the field of spatiotemporal control. For this, we present a novel femtosecond pulse-shaping approach. We explain how to achieve spatiotemporal control by spatially manipulating the system through trapping and subsequently or simultaneously exerting temporal control using shaped femtosecond pulses. By leveraging ultrafast femtosecond lasers, the prospect of having temporal control of molecular dynamics increases, and it becomes possible to circumvent the relaxation processes at microscopic timescales. Optical trapping is an exemplary demonstration of spatial control that results in the immobilization of microscopic objects with radiation pressure from a tightly focused laser beam. Conventional single-beam optical tweezers use continuous-wave (CW) lasers for achieving spatial control through photon fluxes, but these lack temporal control knobs. We use a femtosecond high repetition rate (HRR) pulsed laser to bypass this lack of dynamical control in the time domain for optical trapping studies. From a technological viewpoint, the high photon flux requirement of stable optical tweezers necessitates femtosecond pulse shaping at HRR, which has been a barrier until the recent Megahertz pulse shaping developments. Finally, recognizing the theoretical distinction between tweezers with femtosecond pulses and CW lasers is of paramount interest. Non-linear optical (NLO) interactions must be included *prima facie* to understand pulsed laser tweezers in areas where they excel, like the two-photon-fluorescence-based detection. We show that our theoretical model can holistically address the common drawback of all tweezers. We are able to mitigate the effects of laser-induced heating by balancing this with femtosecond laser-induced NLO effects. An interesting side-product of HRR femtosecond-laser-induced thermal lens is the development of femtosecond thermal lens spectroscopy (FTLS) and its ability to provide sensitive molecular detection.

## 1 Introduction

For a spectroscopist, typical light-matter interactions are kept at perturbative levels, with active attempts to minimize all possible interactions with light so as to recover as much information as possible about the system under study. The experimenter is a passive observer in such studies. However, when one wants to participate in and control light-matter processes, for example, out of a desire to enhance chemical selectivity or reaction yields in photochemical processes, the passive viewer approach of a spectroscopist is not sufficient. The desire to selectively enhance chemical processes gave rise to the dream of “controlling chemistry” (Warren et al., 1993), way back in the 1960s, since the first practical demonstration of lasers.

The most general investigation and manipulation of light-induced processes require simultaneous control over temporal and spatial properties of the electromagnetic radiation on femtosecond time and nanometer length scales (Aeschlimann et al., 2010; Froula et al., 2018; Schmidt et al., 2021). Most vibrational transitions occur within a few femtoseconds, and these are responsible for chemical transformations that make new bonds and break old bonds. The coherent nature of an ultrafast pulsed laser is crucial for manipulating electronic and nuclear motions, and this gave birth to the concept of “coherent control” involving the manipulation of molecular states coherently and, therefore, circumventing the limits of the uncertainty principle for ultrashort laser pulses (Dantus and Lozovoy, 2004). Though coherent control is an exciting principle, it often involves exotic experimental and laboratory conditions with limited success since practical implementations require that we have control and execution within ultra-short timescales. Thus, the most celebrated and successful aspects of control have been under highly specialized circumstances (Hikosaka et al., 2019). For example, in the gas phase, the system is mostly isolated under molecular beam conditions, where a single isolated molecule undergoes the coveted light-matter interaction for the controlled activity, often referred to as the “active control”; or the “passive control” that can be performed in some designer reactions with conditions based on the specific choice of reactants where predictable pathways can be modulated through light (de Vivie-Riedle et al., 2001). We have shown that more generalizability can be achieved for the “active control” methodology with the help of programmable ultrafast pulse shaping approaches. However, under the abovementioned conditions, the requirement of having a single isolated system interacting with light remains important (Kumar and Goswami, 2014).

We propose, in this paper, the idea of a novel laser-directed experimental environment that could work under standard laboratory conditions. This would, therefore, be more of an open laboratory situation where one does not need, e.g., a beam chamber or other isolated environments with ultracold temperatures or specialized conditions for the cold atoms. The idea of generating such situations in a spatially and temporally controlled environment is an interesting mix of the thermal and non-linear processes arising from several aspects, including femtosecond laser interactions. We want to create a programmable and reproducible environment that will still work under relatively open conditions, which forms our basic promise. Though possibilities of using directed and optimal control can also exist (Rice and Zhao, 2000; Arnold, 2017), the approach presented here is distinctly different. We present our approach that uses a pulsed optical tweezers setup for spatiotemporal control, which is different from the usual optical tweezers with continuous-wave (CW) lasers. The inception of pulsed optical tweezers was initiated to alleviate the fact that all CW tweezers have thermal complications destabilizing the trap for extended timescale operations. Currently, this development has led to championing of spatiotemporal control (De and Goswami, 2011), which stems from the fact that it has only recently become a close reality. These include the specific developments taking into account the recent crucial developments in variegated fields of pulsed optical tweezers (De et al., 2009), the understanding and control of thermal aspects even with ultrashort pulses due to their high repetition rates (Bandyopadhyay et al., 2021) as well as rapid pulse shaping demonstration into megahertz timescales (Dinda et al., 2019). These diverse developments have been essential as ultrafast pulsed lasers with varying repetition rates have become critical for light-matter interactions and their control.

As always, the practical limitations of generalized control approaches have been that we have control over processes only within the ultra-short coherent timescales of light-matter interactions (Boyd, 2003). Unfortunately, whenever we go to ultra-short timescales, non-linear interactions are unavoidable. When isolated environments cannot be guaranteed, the additional spatial control knob is crucial, though this begets a non-negligible thermal effect at long timescales (Kumar et al., 2014). We will show how we can convert these vices of thermal effects and non-linearities into virtues. At the very outset, however, for clarity and in keeping with the earlier developments, we begin with the importance of temporal aspects from a control perspective.

## 2 Temporal control

**FIGURE 1:**
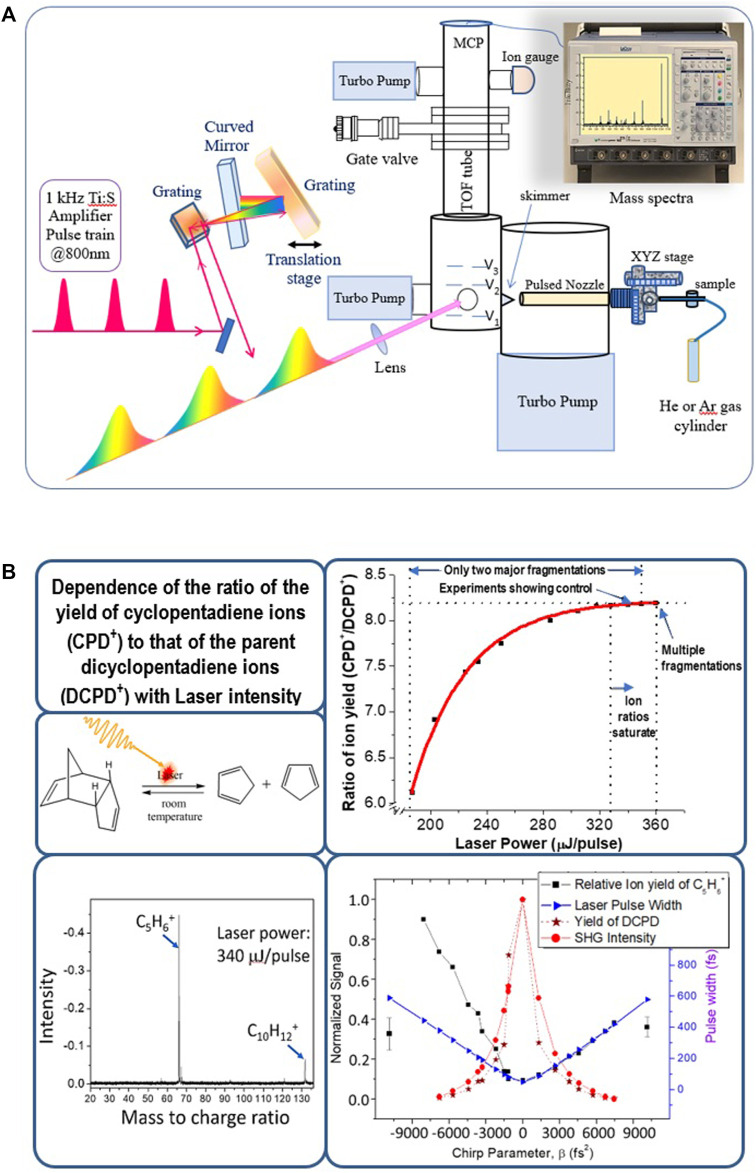
**(A)** Schematic diagram of control experiments with molecular beams with linearly shaped amplified laser pulses. In **(B)**, the specific case of laser photo-fragmentation control of dicyclopentadiene is shown. The data here show that the negatively chirped laser pulses (with negative chirp parameter, β) enhance the fragmentation of dicyclopentadiene to cyclopentadiene.

For practical implementations of temporal control, it is important to use ultrafast laser pulses, preferably in the femtosecond time domain, which pertains to the vibrational period of most molecules. This ensures the photophysical event can occur before the characteristic natural decay timescales. The ultrashort pulses, however, contain large spectral bandwidths making it challenging to generate selective excitations. For example, even a standard commercial 20 fs laser centered at 800 nm has a large bandwidth of ∼47 nm, and its second harmonic at 400 nm will have a bandwidth of ∼23 nm. In fact, an excitation process with ultrashort pulses, though devoid of relaxation complications, will generate a mixture of many states and not a single state, which is often difficult to control. To circumvent this limitation, we devised the approach of selective excitation using linearly chirped pulses under adiabatic conditions. The adiabatic rapid passage principle ensures a robust, smooth selective excitation for isolated molecules (Melinger et al., 1994). Figure 1 shows that even the femtosecond linear pulse shaping scheme can show predictable control based on the sign of the chirp in the fragmentation of dicyclopentadiene to cyclopentadiene under molecular beam conditions (Goswami et al., 2013).

**FIGURE 2:**
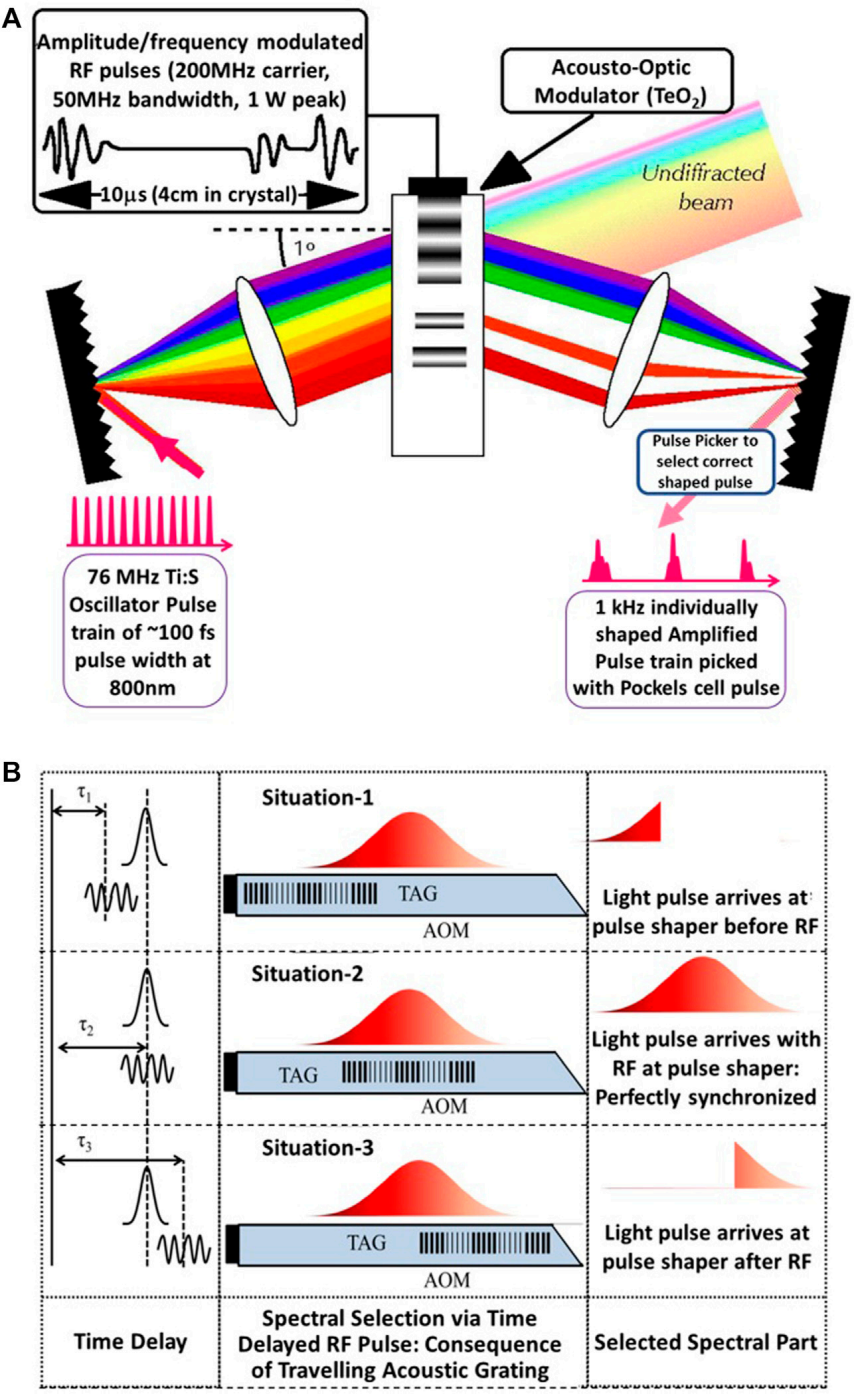
**(A)** Schematic of the Fourier Domain Pulse Shaping approach. For ultrashort pulses, the two lenses can be replaced with curved mirrors. **(B)** Low repetition rate femtosecond pulse shaping in the Fourier domain involves the selection of the desired shaped pulse, which is achieved by the Pockels Cell pulse picker in a 1 kHz amplified pulse shaping process. If needed, each pulse at 1 kHz can have a distinct shape. The control in the Fourier domain is possible by controlling the microsecond radio-frequency (RF) driving the acousto-optic modulator (AOM).

A noticeable improvement in the coherent control scenario involves programmable pulse shaping with a feedback loop, which ensures that control can be attained for isolated molecules (Bergt et al., 2001). Since most individual molecular dynamics are completed within microseconds, a millisecond repetition rate is often ideal. Amplified laser pulse shaping at kHz repetition has thus flourished (Fetterman et al., 1998), and individually shaped pulses at such repetition rates have become routine (Figure 2).

This scenario changes for the case of control in the condensed phase, where under experimental conditions, the principle of an individual isolated molecule interacting with laser cannot be applied. This is because, for condensed matter, unlike molecular beam conditions or low temperature diluted doped crystals, there is always a non-negligible statistical interaction with surrounding molecules. The focus of single-beam optical tweezers is appropriate for spatially controlling their degrees of freedom or their center of mass motion. However, to ensure the possible implementation of simultaneous temporal control, the single-beam optical tweezer setup necessitated augmentations using pulsed lasers resulting in the advancement of pulsed optical tweezers. In this context, we discuss our ultrafast pulsed laser optical tweezers developments.

## 3 Spatial control with ultrafast optical tweezers

**FIGURE 3:**
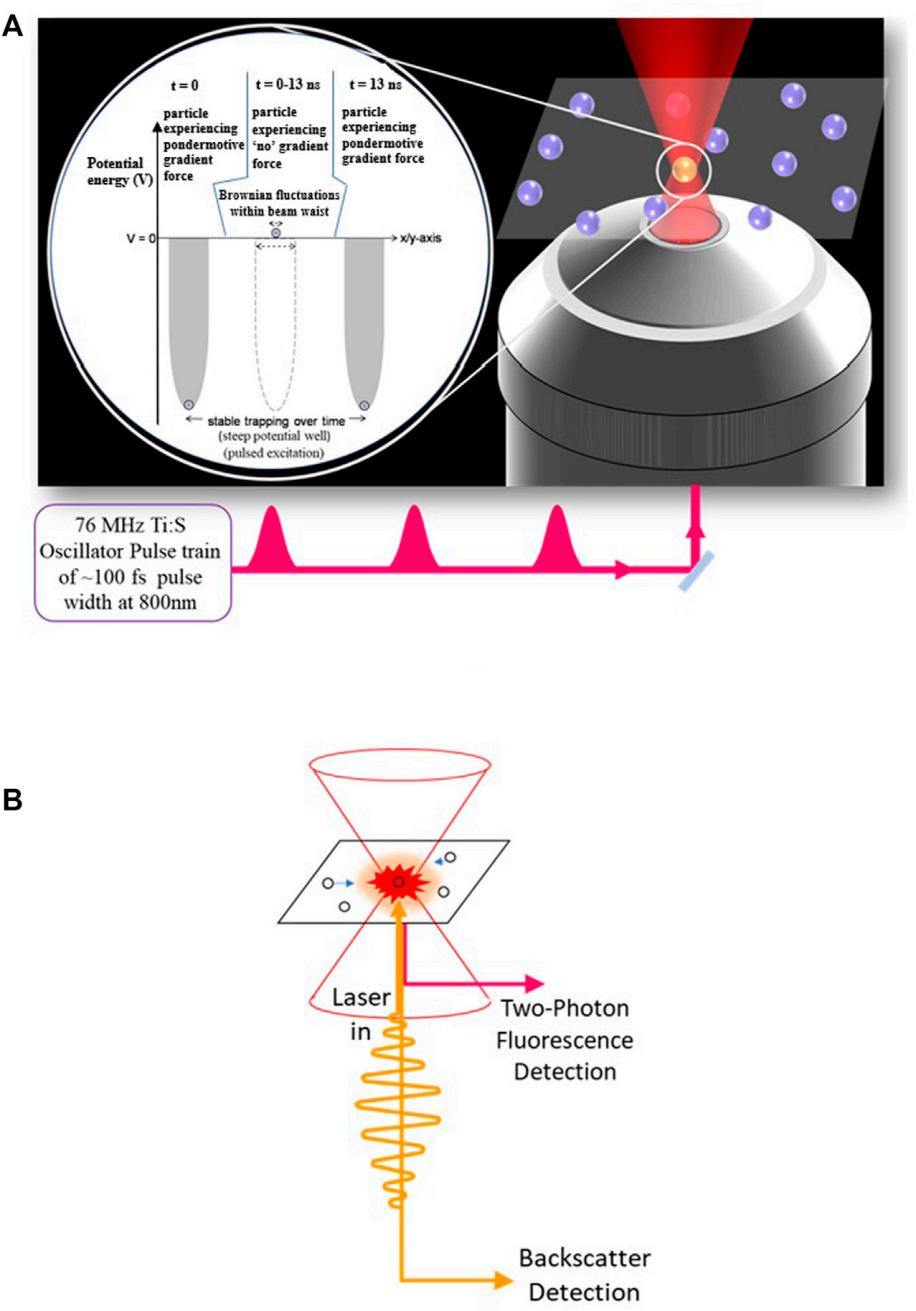
**(A)** Schematic diagram of the Femtosecond pulsed optical tweezers (FOT) and **(B)** its enhanced detection scheme due to the Two-Photon Fluorescence (TPF) detection.

As Arthur Ashkin, (1970) proposed optical tweezers with CW lasers are based on force balancing principles with the laser photon flux, which can be modeled as a Simple Harmonic Oscillator potential well, and calibrated for sensitive measurements accordingly (Ashkin and Ebrary, 2006). The scattering force on the particle can be balanced by the gradient force generated from the photon flux. Such a balance is achieved for a sharply focused Gaussian beam profile, which is the working principle behind CW optical trapping. For a pulsed laser, however, the gradient force will only be present during the pulse and not otherwise. Nevertheless, pulsed laser optical tweezers have worked reliably with high repetition rate (HRR) lasers operating at several MHz frequencies (Figure 3). This is because even the fastest possible naturally occurring process of scattering, the Brownian motion in liquids, would require about a microsecond for the particle to move away from the laser’s focal point. Thus, subsequent pulses from an HRR laser would sample the same object, and the necessary force balancing for optical tweezers remains viable. Optical tweezers are very sensitive to their immediate environments and, in fact, once calibrated, they are very effective in probing microenvironments and any changes thereof with high sensitivity (Goswami, 2015; Mondal and Goswami, 2015; Mondal et al., 2016).

**FIGURE 4:**
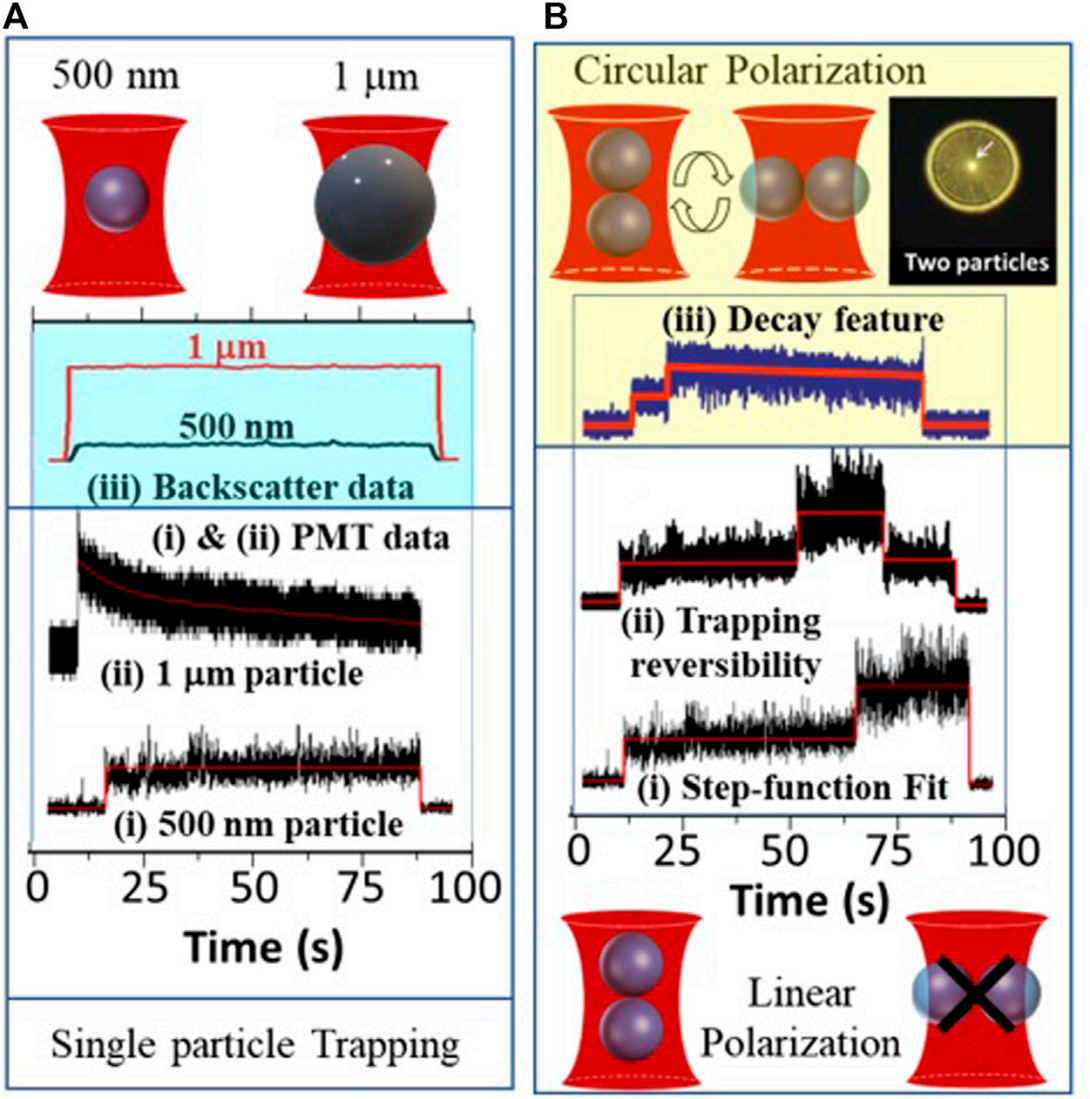
Highlighting the characteristics of FOT: **(A)** Size-dependent TPF signal decay in FOT as shown for (i) 500 nm *versus* (ii) 1-micron fluorophore-coated-bead through photo-multiplier tube (PMT) detection, which shows the importance of total *versus* partial illumination of the tweezed particle. (iii) Same study for backscatter data acquisition does not show any characteristic decay. Given this distinction of FOT, it can be used for determining the orientation of multiply trapped beads: **(B)** Use of linear *versus* circular polarization in the TPF data acquisition (i), (ii), and (iii) respectively, for determining the possible orientation of the two co-trapped 500 nm beads.

An immediate advantage of using ultrashort pulses for optical tweezers lies in the capability of inducing non-linear processes that can enhance the detection of optically trapped particles through, say, the TPF effect (De et al., 2011). Enhanced detection sensitivity with TPF has also enabled the observation of smaller trapped particles because it gives rise to resolution enhancement as it is much smaller than the focusing wavelength (De and Goswami, 2011). As shown in Figure 4, this high signal-to-noise ratio (SNR) with TPF has also enabled the visualization of the optically induced aggregation effects (Mondal and Goswami, 2016; Roy et al., 2016; Roy, Mondal, and Goswami, 2016) in femtosecond optical tweezers (FOT). In fact, FOT is more optimized for Rayleigh particles that behave as point dipoles, which would be the limiting case for approaching the single-molecule domain.

Another critical aspect of ultrafast pulsed optical tweezers lies in their superior management of the thermal elements of optical tweezers (Bandyopadhyay et al., 2021), which we will discuss in detail after covering the spatiotemporal aspects of the ultrafast optical tweezers. In fact, the background advancements discussed above have led us to a discussion on the development of ultrafast pulse shaping at MHz repetition rates which would be critical for implementing simultaneous temporal control with the ultrafast single-beam optical tweezer.

## 4 HRR femtosecond pulse shaping

**FIGURE 5:**
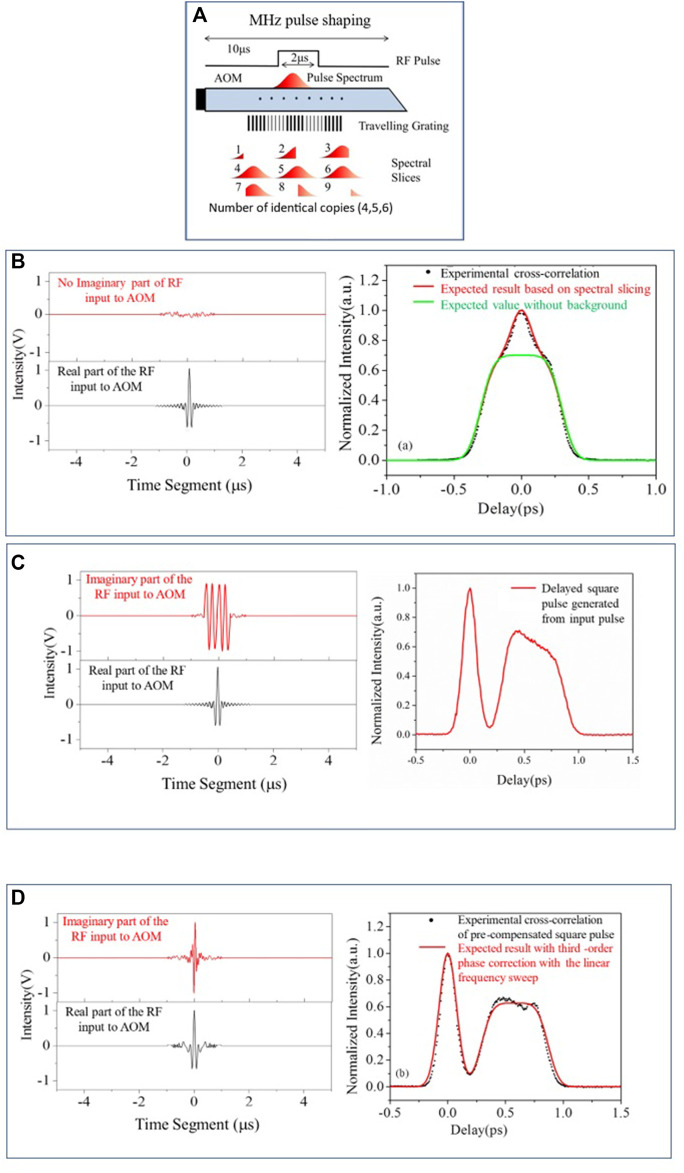
Megahertz repetition rate femtosecond pulse shaping: **(A)** Schematic representation showing that spectral slicing is the key in this MHz pulse shaping scheme in contrast to pulse picking for the low repetition method. **(B)** Since each pulse at 76 MHz cannot be shaped, there is a background of the original pulses (incident from the laser) in this pulse shaping approach. **(C)** Application of the Fourier phase provides the requisite time delay to separate the shaped pulses from the unshaped background. **(D)** Finally, distortion compensation by pre-compensating the RF by adding a third-order phase with the linear frequency sweep, which generates the correct individually shaped square pulses at ∼10 MHz.

Most of the programmable femtosecond pulse shaping approaches are based on Fourier Transform (FT) techniques (Goswami, 2003), as femtosecond timescales are too short for direct time-domain modulations. At a kHz repetition rate, femtosecond arbitrary pulse shaping has been successfully demonstrated by selecting and amplifying the shaped pulses through an AOM (Figure 2), acting as a traveling grating in the Fourier domain (Hillegas et al., 1994). However, it is almost impossible for the HRR femtosecond lasers to generate and select a single “correct” shape through the Fourier approach alone due to the finite refresh rate and transit time of a traveling wave grating through an AOM. Fortunately, since a phase change in the Fourier domain translates to a time delay in the inverse Fourier domain, we used this approach of complex pulse shaping [both amplitude and phase modulation (Yang et al., 1998)] to demonstrate HRR femtosecond pulse shaping at MHz repetition rates. The specific demonstration is shown in Figure 5, where individually shaped pulses at ∼10-MHz repetition rates were generated that were temporarily shifted from the 76-MHz incident laser pulses from the Coherent MIRA 900F^©^ femtosecond Ti:Sapphire laser using the Fourier delay principle (Dinda et al., 2019).

Such MHz repetition rate pulse shaping, as shown in Figure 5, would be suitable for pulsed optical tweezers applications, as mentioned in the previous section. Given this possibility of using shaped pulse FOT, let us now revisit the theoretical aspects of FOT that arise when faced with NLO in addition to the thermal issues of optical trapping.

## 5 FOT: Subtle balancing of thermal effects with NLO effects

**FIGURE 6:**
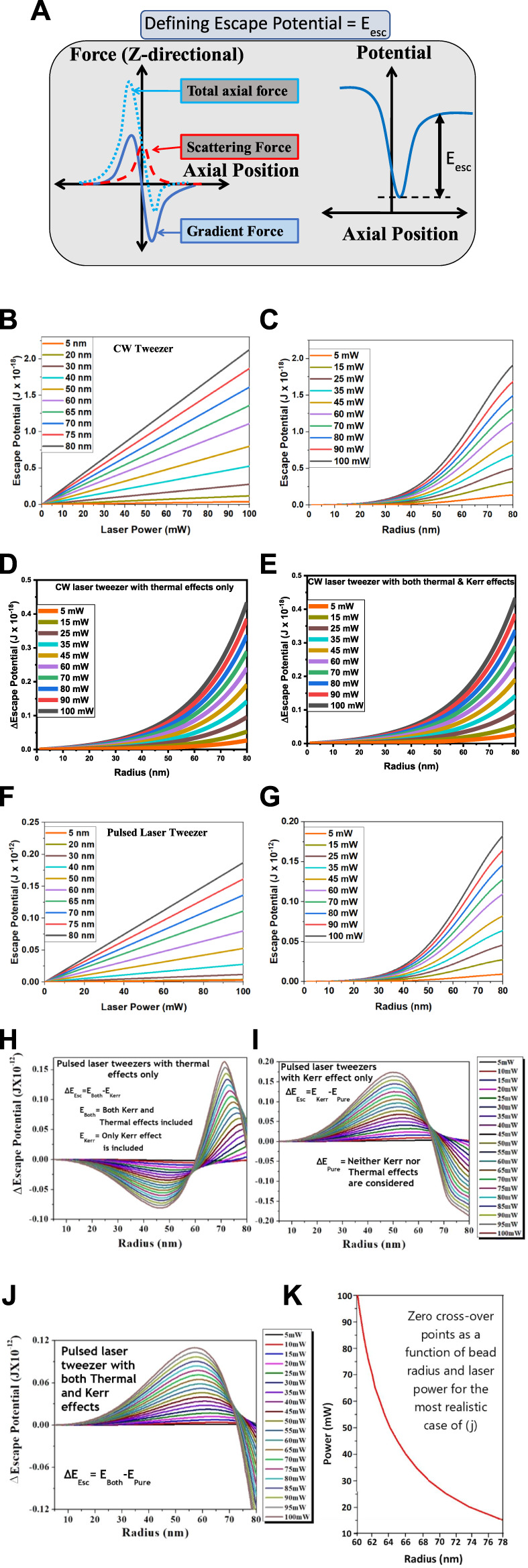
A theoretical model of optical tweezers for both CW and pulsed lasers: **(A)** Schematic of the force balancing used for the calculations. For CW tweezers, as shown in **(B)**, the calculated escape potentials scale linearly with increasing laser powers, which also depends on the trapped bead radius. However, as shown in **(C)**, the trapped bead radius dependence on the calculated escape potentials is not linear. A more useful approach is to use the difference in the escape potentials for **(D)** thermal effects only and **(E)** both thermal and Kerr effects, compared to the case with neither effect present. Note that **(D)** and **(E)** is essentially the same since the Kerr effect is not induced with CW lasers. For pulsed tweezers, **(F)** the calculated escape potentials are about 5-orders in magnitude higher due to high peak powers and still scale linearly with increasing laser powers. **(G)** For the simplest case without any thermal or Kerr effect, the bead radius dependence on the calculated escape potentials is also like that of the CW laser case except for the higher magnitude. However, the dependence of trapping bead radius on the difference in the escape potentials looks much more complicated with **(H)** only the Kerr effect and with **(I)** only the thermal effect compared to the cases when neither effect is incorporated. **(J)** We also have the distinct case where both thermal and Kerr effects are present, which is the most realistic case for femtosecond pulsed laser optical tweezers. **(K)** Finally, since the thermal and Kerr effects often impact in opposite ways on non-linear refractive index, they can balance each other as a function of trapping bead radius.

Thermal problems are omnipresent in optical tweezer experiments, irrespective of whether CW or pulsed lasers are generating them. In general, thermal effects for CW lasers are understood in terms of “thermal lens” generation due to changes in the refractive index arising from the thermally induced heat load (Català et al., 2017). Most systems expand on heating. As a consequence, a reduction of the refractive index occurs. Thus, the thermal lens (TL) is primarily a diverging lens. Due to the high disparity in timescales for a single femtosecond laser pulse, the thermal effect is insignificant. However, for HHR femtosecond lasers, which is the requirement for, say, an optical tweezer, cumulative thermal effects occur as well (Singh et al., 2021). Additionally, the use of femtosecond pulses for optical trapping also invokes NLO processes. Much of the NLO effect induced is a result of the Kerr non-linearity. Interestingly, ultrafast-laser-induced non-linearities can offset thermal effects in femtosecond optical tweezers since the refractive index changes due to TL and the ones due to the Kerr effect have opposite signs for many systems over varying conditions for the laser parameters (Goswami, 2021). This result is of significant benefit (Figure 6) as it is an important aspect of control that can be achieved through the manipulation of several light-matter interactions depending on the tweezing particle’s size and environment as well as the tweezer laser’s characteristics like its beam size and shape, laser pulse width, center wavelength, phase, etc., As shown in Figure 6, we define an important parameter in this context, the “escape potential,” which can effectively be used to quantify the stability of the optical trap.

A further important aspect of this pragmatic developmental approach to control is that it does not ignore or oversimplify the practical ubiquity of thermal effects, which also interfere with NLO measurements; this is evident from the discussion above. There have been multiple attempts to minimize or avoid thermal effects with varying levels of success (Singhal et al., 2017; Maurya et al., 2019). Interestingly, FOT can also measure with high precision at micron resolution the impact of laser-induced thermal effects that result in temperature and viscosity changes (Mondal, Mathur, Goswami, 2016). For studying a perfectly uniform system, it is best to use a sufficiently rapid moving or flowing design to circumvent thermal effects by effectively regenerating the sample. However, the most important fallout of this recognition of thermal effects, even with femtosecond lasers at HRR, is perhaps the development of femtosecond thermal lens spectroscopy (FTLS) (Bhattacharyya et al., 2010), which we will discuss next.

## 6 The FTLS: Transforming vice to virtue

**FIGURE 7:**
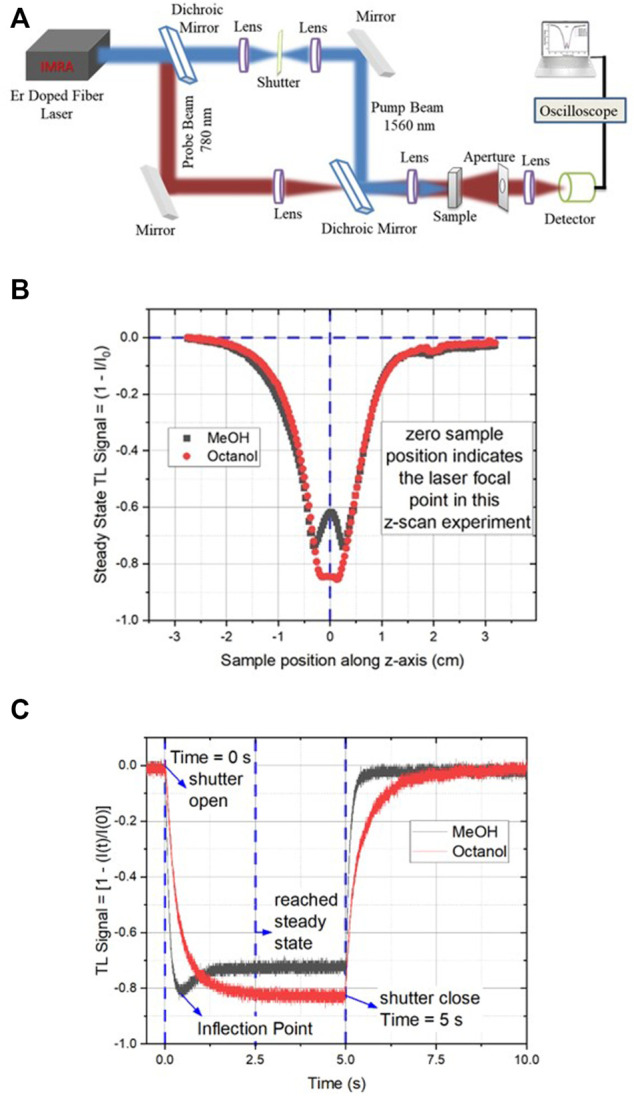
Femtosecond thermal lens (TL) spectroscopy with dual laser beams: **(A)** Experimental setup for both stationary as well as time-resolved TL measurements with high-repetition-rate lasers in a dual-beam experimental configuration. **(B)** Typical steady-state TL signals for dual-beam experiments for two different samples were collected as a function of sample position across the focal point of the TL-inducing laser. Drastic molecular dependence of TL is evident between methanol and octanol. **(C)** The corresponding time-resolved TL signal shows the molecular distinction in femtosecond TL. The inflection point indicates the importance of convection effects in addition to the conduction models that are molecule insensitive.

**FIGURE 8:**
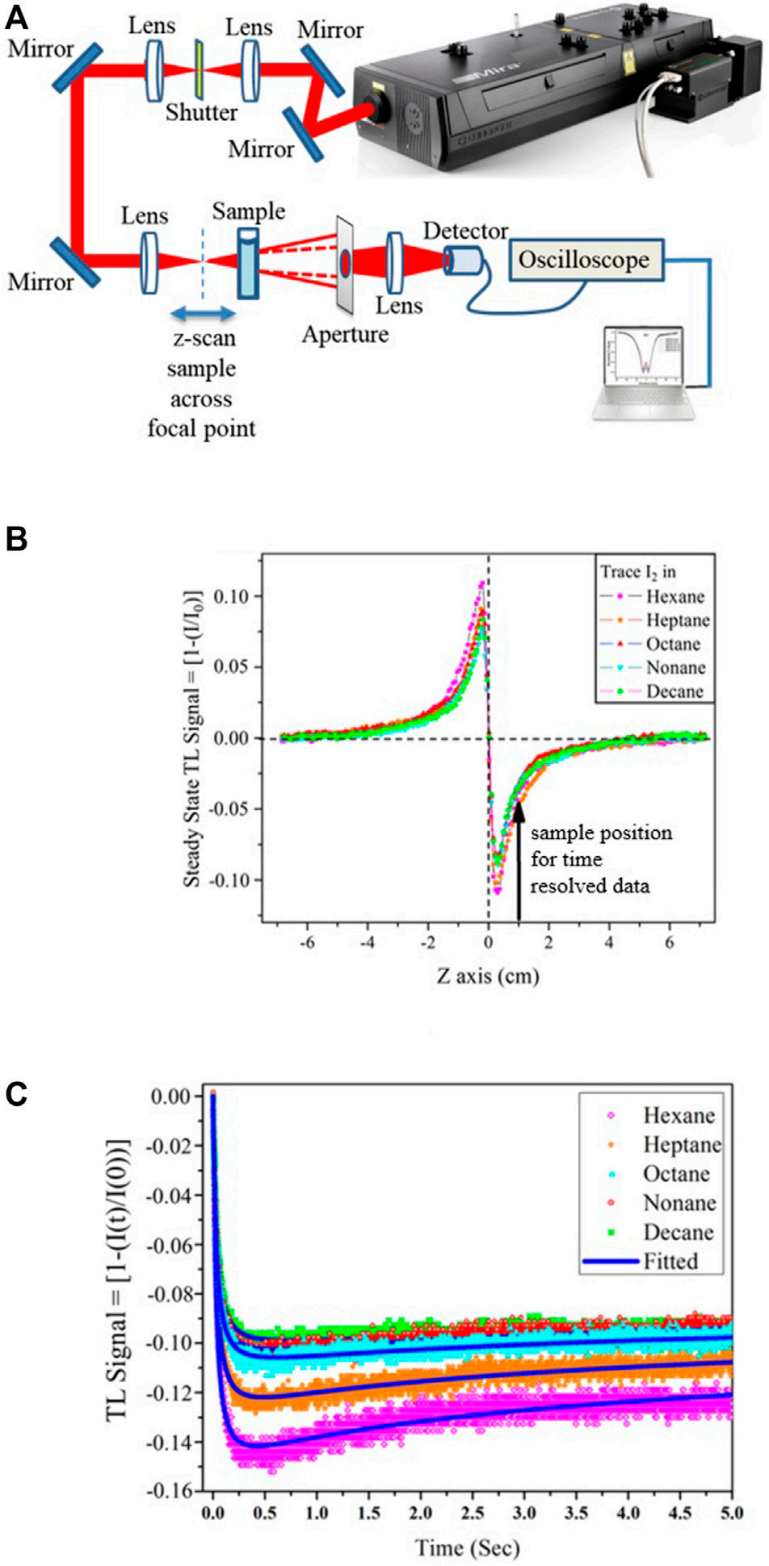
Femtosecond thermal lens (TL) spectroscopy with single laser beam: **(A)** Experimental setup for both stationary as well as time-resolved single beam femtosecond TL studies. **(B)** Typical steady-state TL signals for single-beam experiments as a function of sample position across the focal point of the TL-inducing laser for various samples showing zero-crossing at the focal point. For time-resolved TL collection, a position other than the focal point indicated by the arrow is chosen. **(C)** Time-resolved TL signals for Single beam femtosecond TL studies for the same shutter opening window. The early-time diagnostics of the shutter open part shows the importance of early time versus steady state. Note that for alkane samples, where the thermal effect is low, I_2_ was added to keep the extent of TL generation steady across different samples. Fitting of the data is possible with Eq. 2, as discussed in the text.

In general, thermal effects always interfere with the measurement process. However, there are circumstances like the one presented in the previous section where thermal effects may be beneficial to offset NLO effects in femtosecond optical tweezers. Similarly, FTLS has been developed and proven to be an incredibly versatile technique (Bhattacharyya et al., 2010; Singhal and Goswami, 2019). Though each femtosecond laser pulse provides an almost negligible energy load to the sample, the cumulative effect of HRR femtosecond pulses results in a significant thermal load (Kumar et al., 2014). Such an accumulative effect with minuscule heating capability is even suitable for highly volatile systems and a smooth transition into out-of-equilibrium conditions without other delirious effects under reasonable experimental conditions (Kumar et al., 2014). The heat load generated is maximum at the center of the beam resulting in a temperature gradient leading to the creation of a refractive index gradient and results in the “thermal lens,” a lens-like optical element in the sample (Leite et al., 1964; Roess, 1966; Osterink and Foster, 1968; Foster and Osterink, 1970). Subsequently, various heat dissipation dynamics arise, *viz.*, thermal conduction, convection, and radiative relaxation, all of which work together to equilibrate the heat generated in the sample. Amongst these, thermal convection plays a significant role for fluids primarily associated with molecular movement and is, thus, found to be strongly correlated to molecular properties. Previous studies involving continuous irradiation focused on thermal effects of small magnitudes, where it sufficed only to consider the conductive dissipation effects (Shen et al., 1992; Shen et al., 1995; Marcano et al., 2002; Marcano et al., 2006) to be able to derive exhibited phenomenological and bulk characteristics. The inclusion of thermal convection for ultrafast laser thermal processes, which correlates with molecular properties, has driven the development of ultrafast laser-induced thermal processes for sensitive spectroscopy (Figure 7, 8). Figure 7 represents the dual beam high repetition rate experimental setup where both the steady-state and time-resolved experiments can be carried out.

The experimental data collected can be effectively modeled and fitted by understanding the temperature distribution in space and time created as a result of the excitation laser heat deposition and dissipation. The uniqueness of the FTLS lies in the cumulative nature of the heat deposition process, as discussed.

A single laser beam high repetition rate experimental setup with high sensitivity has, in fact, also been established (Figure 8), where both the steady-state and time-resolved experiments can be carried out (Singhal and Goswami, 2020). The crucial aspect in the experimental design of the single beam technique (Figure 8A) lies in realizing that the stationary sample position for time-resolved measurements cannot be the focal point of the TL-generating laser. This is because there is no TL signal at the focus, so we choose the stationary sample position for time-resolved measurements as indicated by an arrow in Figure 8B, which is quite unlike the dual beam setup.

The TL model that includes both the conduction and convection terms involves a coupled differential equation that can be solved using the dimensionless parameter known as the Peclet number, $P_{E}$ , which is the ratio of convective to conductive heat transfer rates (Bergman, 2019), where the laser beam size, *ω*_e_, itself imposes the characteristic TL length. Mathematically,

$$Peclet\;Number\;\left(P_{E}\right)=\frac{Rate\;of\;convection}{Rate\;of\;conduction}=\frac{\omega_{e}v_{x}}{4D}$$ (1)

where, *D* is the thermal diffusivity and *v*_*x*_ is the convection velocity through the region of investigation. This can result in the TL fitting model that uses both conduction and convection (Singhal and Goswami, 2020):

$$TL\;Signal={\left|1-\frac{j\theta }{4}\left[\begin{array}{c} -\left(1+\frac{P_{E}^{2}}{2}\frac{{\left(1+jV+2m\right)}^{2}}{2m\left(1+jV\right)}\right)\left(2j\tan^{-1}\left\{\frac{2mV}{\left[{\left(1+2m\right)}^{2}+V^{2}\right]\left(t_{c}/2t\right)+1+2m+V^{2}}\right\}+\ln\left\{\frac{{\left[1+2m/\left(1+2t/t_{c}\right)\right]}^{2}+V^{2}}{{\left(1+2m\right)}^{2}+V^{2}}\right\}\right)\\ -P_{E}^{2}\left[\frac{2m}{\left(1+jV\right)}\ln\left(1+\frac{2t}{t_{c}}\right)+\left(\frac{2t/t_{c}}{1+2t/t_{c}}\right)\right] \end{array}\right]\right|}^{2}$$ (2)

The real nature of the TL signal arises from the mod square as shown in the above complex expression involving: $j=\sqrt{-1}$ . The absorbance coefficient, *α*, and the beam divergence angles are small for the propagating laser with wavevector, *k*, and wavelength, *λ*_*p*_. This allows us to consider the exciting laser beam power and beam radius, *ω*_*e*_, to be taken as constant within the cell of path length *l*. The phase change of the collimated probe laser beam, which could be the same as the exciting laser beam, results in the generation of the TL due to a variation in the refractive index $\left(dn/dt\right)$ of the medium (Sheldon et al., 1982; Shen et al., 1992). Thus, Eq. 2 has fitted the experimental data well for the experimental results as shown in Figure 8C. If the exiting and probe beams are not the same, the probe beam is collimated and overfills the exciting beam. We define the parameter, *m*, to be related to the ratio of the two beam waists as: $m=\left(\omega_{1p}^{2}/\omega_{e}^{2}\right)$ . Consequently, the terms: $\theta =-P_{e}\alpha ldn/dt/k\lambda_{p}and\;t_{c}=\omega_{e}^{2}/4D$ *,* relate to different physical properties of the sample responsible for the formation of a thermal lens in the laser interaction volume *V* in such a way that its magnitude is directly related to the strength of the thermal lens.

When $P_{E}=0$ , Eq. 2 reduces to the Shen et al., (1992) equation, which corresponds to situations arising from considering only the conductive mode of heat transfer as below:

$$TL\;Signal={\left[1-\frac{\theta }{2}\tan^{-1}\left(\frac{2mV}{\left[{\left(1+2m\right)}^{2}+V^{2}\right]\left(t_{c}/2t\right)+\left(1+2m+V^{2}\right)}\right)\right]}^{2}+{\left[\frac{\theta }{4}\ln\left\{\frac{{\left(1+2m/\left(1+2t/t_{c}\right)\right)}^{2}+V^{2}}{{\left(1+2m\right)}^{2}+V^{2}}\right\}\right]}^{2}$$ (3)

Most literature involving the Shen model has a very low heat load. Therefore, the first term of Eq. 3 suffices, which can then be reduced to the popular mathematical expression for the Shen model (Shen et al., 1992; Shen et al., 1995) given by:

$$TL\;Signal={\left[1-\frac{\theta }{2}\tan^{-1}\left(\frac{2mV}{\left[{\left(1+2m\right)}^{2}+V^{2}\right]\left(t_{c}/2t\right)+\left(1+2m+V^{2}\right)}\right)\right]}^{2}$$ (4)

Thus, FTLS can distinguish between molecules within fluids based on both size and shape (Kumar et al., 2014; Kumar et al., 2014). Similarly, the differentiation of isomers and isotopes in fluids is also effectively possible with FTLS (Bhattacharyya et al., 2014; Kumar et al., 2014). Additionally, FTLS is also sensitive to changes in intermolecular interactions, which would lead to phase separations (Goswami et al., 2019), interfaces (Singhal and Goswami, 2020), and several other physical interactions (Maurya et al., 2016; Rawat et al., 2021). Cumulative thermal processes fall in the domain of weak perturbations that do not require strong absorption or any exact resonant excitation conditions. Consequently, they work over a broad range of wavelengths as well as laser pulse widths. Thus, FTLS is an astoundingly versatile technique (Goswami et al., 2021), which is continuously being unveiled to date.

## 7 Conclusion

We have presented the pragmatic aspect of intense laser-induced light-matter control that can circumvent practical experimental constraints. The concept of spatiotemporal control that simultaneously manipulates the position and time dynamics has been discussed in the context of femtosecond optical trapping and is further enabled through the Megahertz repetition rate femtosecond pulse shaping. While the presence of non-linear interactions with femtosecond lasers has been shown to offset the omnipresent thermal effects in optical tweezers, it has also been shown to attain a better signal-to-noise ratio through two-photon fluorescence detection of trapping. As an additional outcome, we have demonstrated how the omnipresent thermal effects can be turned around for cumulatively accumulating minuscule femtosecond induced heat load into an interesting femtosecond laser thermal lens setup for Megahertz repetition rate femtosecond lasers and provide sensitive molecular detection. Such developments in spatiotemporal control with intense femtosecond optical pulse shaping approaches thus promise myriad applications with continuous advancements.

## Data Availability

The original contributions presented in the study are included in the article/Supplementary Material, further inquiries can be directed to the corresponding author.
